# Combined plasma lens and rephasing stage for a laser wakefield accelerator

**DOI:** 10.1038/s41598-024-78143-6

**Published:** 2024-11-01

**Authors:** Cornelia Gustafsson, Erik Löfquist, Kristoffer Svendsen, Andrea Angella, Anders Persson, Olle Lundh

**Affiliations:** Department of Physics, Lund University, P.O. Box 118, Lund, 22100 Sweden

**Keywords:** Optics and photonics, Physics, Plasma physics

## Abstract

Electrons from a laser wakefield accelerator have a limited energy gain due to dephasing and are prone to emittance growth, causing a large divergence. In this paper, we experimentally show that adjusting the plasma density profile can address both issues. Shock-assisted ionisation injection is used to produce 100 MeV quasi-monoenergetic electron bunches in the primary part of the accelerator. Downstream from the accelerator, a second, independently tuneable density region is added, which can be used to either boost the energy of the electron bunches or as a plasma lens for significant divergence reduction. An additional energy gain of 25% and a 40% divergence reduction are obtained. Theoretical models validate the effects.

## Introduction

A Laser Wakefield Accelerator (LWFA)^1^ can deliver electron bunches with energies in the range of hundreds of MeV over an acceleration length of just a few millimetres. The unique properties of the produced electron bunches and subsequent x-ray radiation, together with the compactness of this accelerator, have led to increased interest in further development over the past decades. In its most simple form, a short-pulse laser of high intensity, typically on the order of 10$^{19}$ W/cm$^2$, is focused into a gas of low Z-number (usually hydrogen or helium), which becomes completely ionised, thus forming a plasma. As the laser pulse propagates through the plasma, a non-linear plasma wave is resonantly excited and accelerating field gradients of several hundreds of GV/m are reached^2^. Some electrons gain a momentum so high that they can break free from the collective plasma motion and get exposed to these gradients, i.e., become injected into the accelerating structure of the wakefield. By matching the plasma density to the laser power^3^, it is possible to generate quasi-monoenergetic electron bunches^4–8^. However, this process of injection is sensitive to both plasma and laser inhomogeneities, thus producing electron bunches with fluctuating peak energy and energy spread. Therefore, tremendous efforts have been made to control the injection mechanism, both by manipulating the plasma and the laser parameters. Another limitation of the LWFA is the difference in group velocity between the laser pulse and electron bunch. The laser pulse, as it propagates through the plasma, has a group velocity less than the speed of light in vacuum, *c*. The electrons within the bunch, on the other hand, are highly relativistic and thus have a speed that is very close to *c*. The electron bunch will therefore, after a certain propagation length, be exposed to the decelerating phase of the wakefield, which is defined as half-of the plasma period^3^. This phenomenon is called dephasing and is one of the greatest limitations to the maximum achievable particle energy of the LWFA. This work combines several methods to produce quasi-monoenergetic electron bunches with a few milliradians of transverse divergence. The energy of these electrons is also boosted.

An injection technique proven robust is the so-called shock-assisted ionisation injection^9^, which combines shock-front injection^10,11 ^and ionisation injection^12^, utilising a gas mixture of helium doped with nitrogen. The K-shell electrons of nitrogen are ionised as the peak intensity of the laser pulse traverses the shock-front region. Shortly after the density perturbation, as the plasma density decreases, the electrons are trapped, while further ionisation and trapping are mitigated due to the plasma wake expansion. Since the shock region is only a few micrometres wide and the density transition between the shock and background plasma density is steep, the injected electrons are well localised within the accelerating structure, leading to electron bunches of low energy spread. Effectively, density manipulations at the beginning of the acceleration dictate the longitudinal properties of the accelerated electron bunch.

**Fig. 1:**
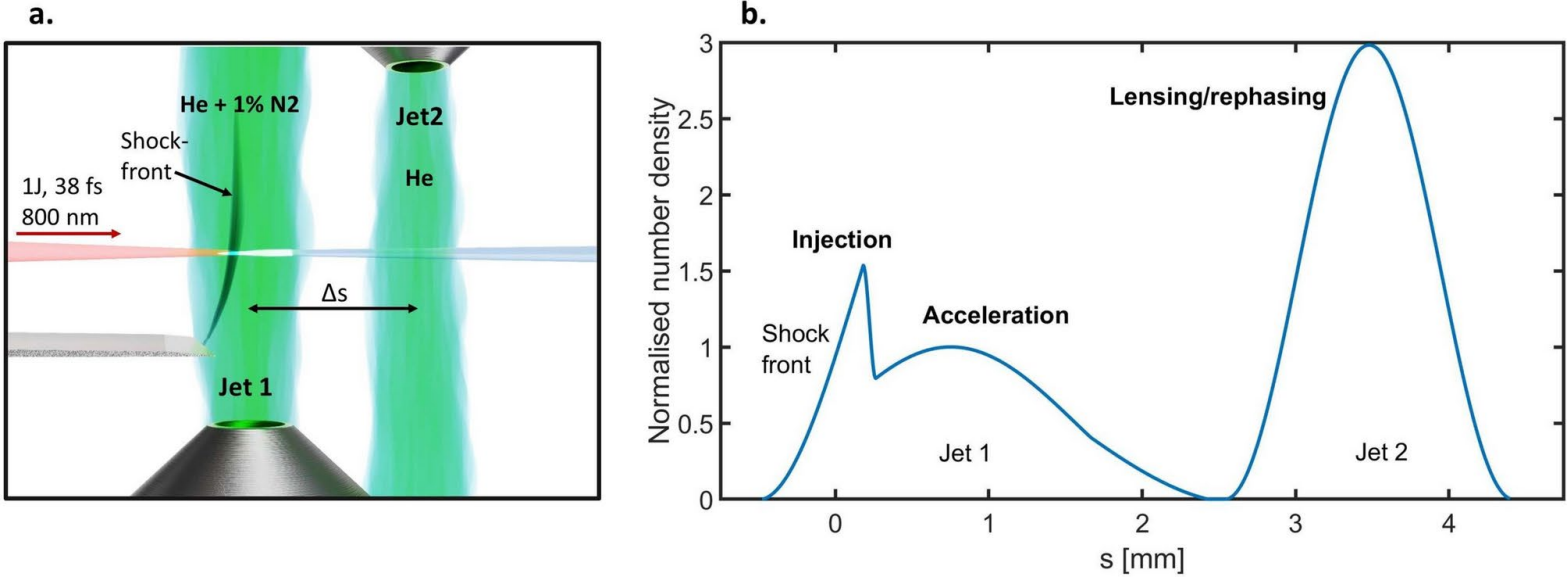
A schematic of the experimental arrangement in (**a**) and in (**b**) the reconstructed density profile, estimated from interferometric measurements. $\Delta s$ refers to the centre-centre distance between the two jets. In (**b**), the plasma density, $n_\text {e}$, in each region is shown normalised to the peak density of the acceleration region of Jet 1.

The transverse wakefields focus the electrons toward the laser axis (longitudinal axis). The energy of the electrons and their radial displacement at the event of the injection influence the transverse divergence, which can be as large as tens of milliradians. An efficient way to reduce the transverse divergence is by using a plasma lens^13–18^. In this work, a plasma lens operating in the overdense regime is used^19^, such that $n_\text {e} \gg n_\text {b}$, where $n_\text {e}$ and $n_\text {b}$ are the electron density in the background plasma and bunch, respectively. The lens itself consists of a low Z-number gas, in this case, helium, which is easily ionised by the diffracted drive laser. As the electron bunch propagates through the plasma lens, the space charge is shielded by the plasma ions. The net force experienced by the bunch is due to the self-generated azimuthal magnetic field, such that the radial Lorentz force is $F_\text {r}\approx e^2n_\text {b}/(2\epsilon _0)r$, where *r* denotes the radial coordinate, *e* the elementary charge, and $\epsilon _0$the vacuum permittivity. Since the plasma can sustain extreme field strengths, a plasma lens can be made as compact as a few millimetres. A plasma lens is thus able to meet the requirements on size, transverse focusing field strength for highly relativistic particles, and supports chromatic particle beams^15,16^.

If the plasma lens is placed close to the primary accelerator, such that the electrons enter the second plasma region almost immediately after the primary accelerator, and the laser intensity is still such that a weakly non-linear wake can be excited, then the electrons are, in principle, never leaving the accelerating structure. The laser pulse, which has undergone relativistic pulse compression^20^, will resonantly drive a wake in the second density region, in which the electrons can be further accelerated. For maximum energy gain, the plasma density should be increased at a position where the electrons have almost dephased in the main part of the accelerator. This is called rephasing^21,22 ^since the electrons are found yet again in an accelerating phase of the LWFA. However, even if the electrons have not yet reached dephasing, the increase in density boosts the particle energy^21^, if the increase of plasma density leads to a wake contraction such that the electron bunch remains in an accelerating phase. In this paper, we present experimental results that combine the plasma lens and the rephasing stage in one single setup.

## Method

A schematic of the setup is shown in Fig. 1a, where Jet 1 is the accelerator and Jet 2 is the plasma lens and rephasing stage. The experiment is conducted at Lund Laser Centre using the multi-TW Ti:Sapphire CPA laser system, linearly polarised in the horizontal plane. The 1 J, 38 fs, and 800 nm centre wavelength laser pulse is focused with an f/13 off-axis parabolic mirror to a full width at half-maximum (FWHM) focal spot size of $w_0 = 12$ $\upmu$m into a 1.5 mm in diameter helium + 1% molecular nitrogen supersonic gas jet. The estimated normalised vector potential is $a_0 =2.7-2.8$, assuming a spatially and temporally Gaussian laser pulse. Jet 1 is positioned 2.8 mm below the laser axis. A razor blade is placed at the beginning of Jet 1, transverse to the laser propagation direction, 2.0 mm above the nozzle opening, and can be translated along the laser propagation axis. The blade perturbs the plasma density as depicted in Fig. 1b, creating a shock-front, where the electrons are trapped at the transition between the shock-front and the plateau. In the figure, the shock-front is marked as “injection” and the plateau as “acceleration”. For the experimental conditions presented, the shock typically has a density between $1.5 - 3$ times the plateau density, as depicted in Fig. 1b. At the beginning of the experiment, the shock position and density of the first jet are optimised to generate a stable, peaked electron spectrum.

The electrons from Jet 1 propagate together with the laser to Jet 2, which is a 1.0 mm-diameter helium supersonic gas jet placed 1.5 mm above the laser axis, such that Jet 1 and Jet 2 have opposing orifices. Helium is chosen as the target gas of Jet 2 because the laser pulse is still able to fully ionise this gas, but the intensity of the laser pulse after Jet 1 is low enough to mitigate self-injection in Jet 2. The jet separation and density in Jet 2 are varied during the experiment, investigating the effect of the second jet. For all measurements made, the configuration of the first jet is kept constant.

After the two jets, approximately 30 mm downstream, a 0.83 T dipole magnet is placed such that the electrons are dispersed in the horizontal plane onto a scintillating screen, which is used to characterise the electron energy spectrum, with the lowest detectable energy at 15 MeV. The screen is imaged with a 16-bit CMOS camera. The electron bunch transverse divergence is obtained from the electron spectrum and is thus measured transversely to the deflection and polarisation plane. Both the reference divergence and the reduced divergence are measured as the FWHM transverse size of the electron bunch.

The plasma density is probed transversely to the plasma channel, where the probe beam is obtained by intercepting the wing of the main laser pulse and redirecting it such that it intersects the interaction area transverse to the main pulse. The probe propagates through a 200 $\upmu$m pinhole to reduce the laser intensity before the gas jets and expand the beam such that the whole laser-plasma interaction region is illuminated. The laser pulse is sent to a Mach-Zehnder interferometer, and the interferogram of the plasma channel is imaged on a 12-bit CMOS camera. The phase shift is retrieved by Fast Fourier Transform, and the density is retrieved through Abel inversion. Fig. 1b shows the best fit of the normalised retrieved plasma density when the centre-to-centre distance between the jets is 2.7 mm.

## Results and discussion

### Injection and trapping

**Fig. 2:**
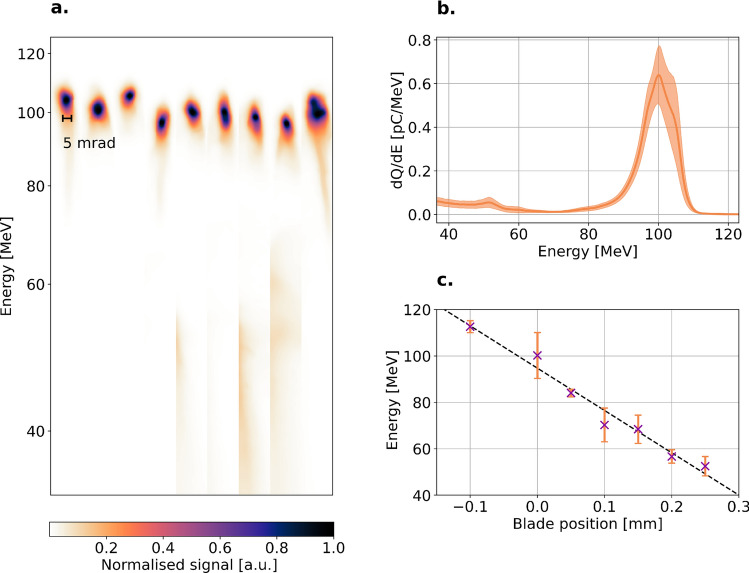
Stable injection from shock-assisted ionisation injection. (**a**), the electron spectrum of 9 consecutive shots at a razor blade position of 0.0 mm, which is precisely at the opening of the orifice of the 1.5 mm jet. The peak density of the plateau is kept at $2.7\times 10^{18}$ cm $^{-3}$. Marked in the figure, is a reference for 5 mrad in the collected spectra and shows the direction over which the divergence is measured. In (**b**), the mean charge density electron spectrum of the consecutive shots in (**a**). The shaded area is the standard error of the mean. In (**c**), the bunch peak energy with respect to the razor blade position, where an increasing position is toward the centre of the jet. The dashed line is a linear fit to the experimental data (crosses, with error bars showing the r.m.s. deviation), effectively giving an accelerating gradient of approximately 180 GV/m.

The injection mechanism is studied by leaving Jet 2 in Fig. 1 off. By characterising the electron bunches from the accelerator alone, a reference of typical electron bunches from the shock-assisted ionisation for our setup is obtained. The electrons in Fig. 2 have an energy spread of 10 MeV FWHM, with a standard deviation of 1.1 MeV and a typical transverse FWHM divergence of 6 mrad. In Fig. 2a, the marking indicates a 5 mrad FWHM transverse divergence. In Fig. 2b, the mean electron spectrum and the standard error of the mean are shown. The razor blade can be translated along the laser propagation direction, adjusting the shock position. Consequently, the acceleration length and the peak particle energy can be tuned. In Fig. 2c, the blade position refers to the position along the longitudinal axis, where the 0.0 mm position refers to the edge of the nozzle orifice of Jet 1. The tuneability of the acceleration length allows a determination of the effective longitudinal field strength within the wake, which is found to be approximately 180 GV/m. At about 2 mm above the nozzle orifice, the gas flow has expanded somewhat, and a blade position outside of the nozzle orifice (-0.1 mm in Fig. 2c) is still able to produce a shock. At this position, by the edge of the nozzle, the free expansion of the gas leads to a gas velocity higher than that in the centre. A sharp shock front can therefore be created at these regions since the high Mach number leads to a narrow shock. As the blade is moved towards the centre of the jet, the shock properties change as the Mach number of the gas decreases^23^. To maintain a high bunch quality and a high particle energy, it is therefore beneficial to create a shock at the edge of the orifice of the jet. Another requirement for a well-defined electron bunch is that the plateau density is significantly lower than that of the perturbation, such that any further injection is mitigated. For a plateau density of $2.7\times 10^{18}$ cm$^{-3}$ in Jet 1, and with the assumption that the laser pulse evolves minimally while traversing Jet 1, the dephasing length (using the formulation from Lu *et al.*^3^) is estimated to be approximately 4 mm. Hence, both from Fig. 2 and theoretically^21^, dephasing is not reached across the plateau. The low density of the plateau alleviates the effect of dephasing because the group velocity of the laser is less affected since $v_\text {g}/c\approx 1-n_\text {e}/2n_\text {c}$ if $n_\text {e}<n_\text {c}$, where $n_\text {c}$is the critical plasma density. However, at low plasma densities, the plasma is not able to balance laser pulse diffraction. As a consequence, the laser pulse is not efficiently self-focusing and the accelerating gradient of the wakefield will decrease, which limits the maximum energy gain. Both effects can be mitigated by increasing the density at the end of the acceleration^21^, as will be discussed later in this paper. For the succeeding results, the razor blade is kept at 0.0 mm and the peak density of the plateau at $2.7\times 10^{18}$ cm $^{-3}$, leading to a stable generation of 100 MeV electrons.

**Fig. 3:**
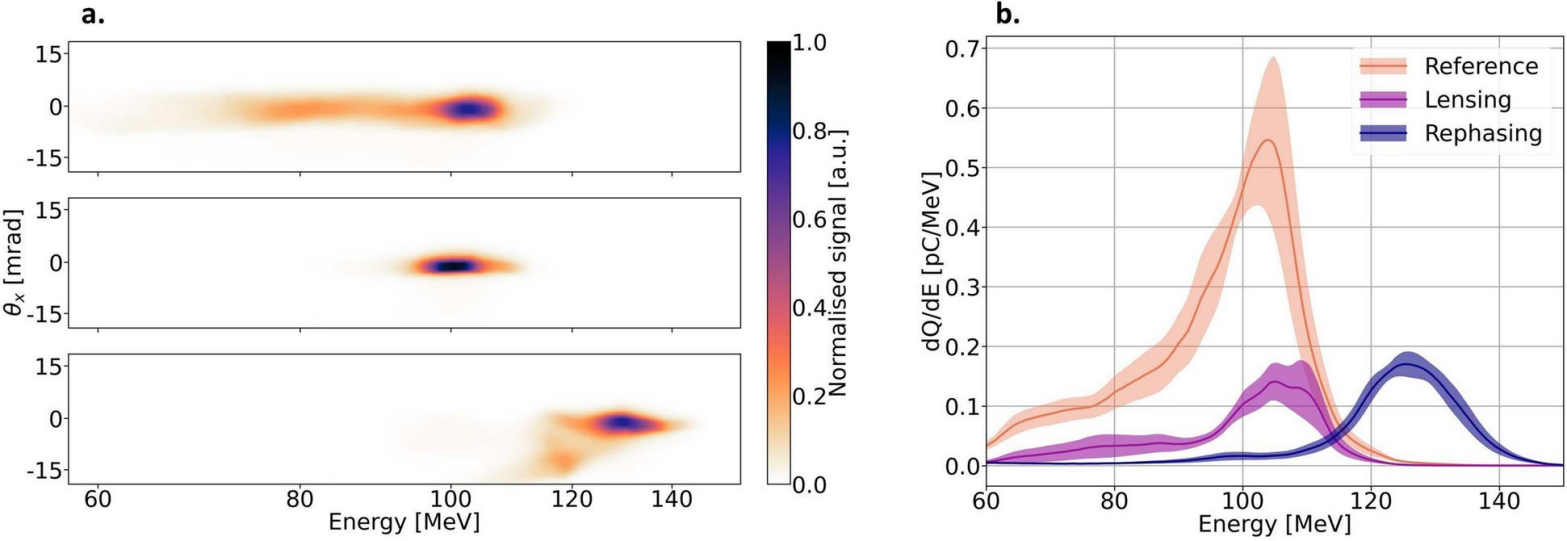
Jet 2 optimised for different operational modes. In (**a**). the top figure shows a typical spectrum when Jet 2 is off. The middle figure with Jet 2 on, optimised for lensing at the detector, and the bottom figure is when Jet 2 is configured such that maximum energy boost is obtained, i.e., it is optimised for rephasing. In (**b**), the mean spectrum for five consecutive shots for each configuration. In orange, the reference spectrum for when Jet 2 is off, purple, Jet 2 optimised for lensing, and blue energy boost. The shaded area shows the sampling distribution of the spectrum.

### Lensing and rephasing

When Jet 2 is on, an additional region of plasma is added downstream from the primary accelerator (Jet 1), as shown in Fig. 1b. The electron bunches from the shock-assisted ionisation injection scheme typically already have low divergence; as stated in the previous section, this is approximately 6 mrad. The divergence is influenced by the decrease in plasma density between $s=0.8$ mm and $s=2.5$ mm in Fig. 1b, essentially contributing to a downramp at the end of the acceleration^24–26^. A downramp may mitigate the emittance growth of the electron bunch, since the focusing strength of the wake decreases with decreasing plasma density^26^. The properties of the gradient, and subsequently the divergence, are not straightforward to control for the configuration presented here. As discussed in the previous section, the maximum energy gain in this configuration is limited due to the decrease in plasma density after the shock front. By adding the second jet (Jet 2), an extra degree of freedom is introduced with respect to beam control. Both the plasma density, $n_\text {e}$, and the jets’ centre-to-centre separation, $\Delta s$, can be chosen arbitrarily, and Jet 2 can thus be used either as an overdense plasma lens or as a rephasing stage.

Due to the low density of Jet 1’s plateau, it is assumed that the laser pulse, with a Rayleigh length of $z_\text {R}\approx 400$ $\upmu$m (see Method for details), has diffracted such that its intensity is below the intensity required for self-injection in Jet 2. The presence of a plasma channel in Jet 2 could be detected both on interferograms and shadowgrams, probed transversely to the laser pulse direction of propagation. The setup is similar to the case of a hybrid LWFA and electron-driven plasma wakefield accelerator (PWFA)^27^, but with the difference that the electron bunch from Jet 1 is interacting with a plasma and a linear wake in Jet 2.

**Fig. 4:**
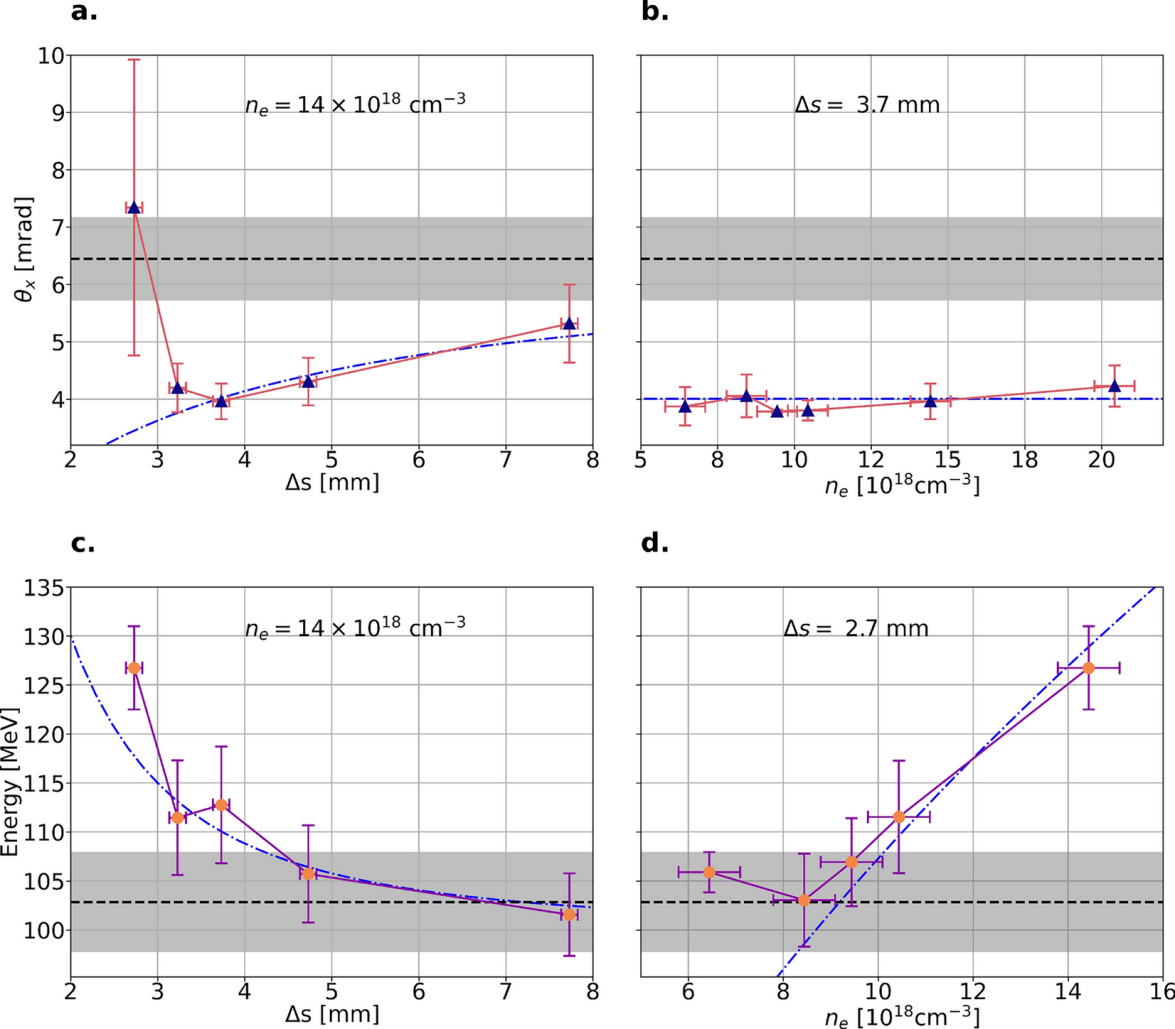
The effect of Jet 2 on the electron bunch generated in Jet 1. In (**a**) and (**b**), the effect on the transverse divergence, $\theta _\text {x}$ with respect to the jet separation, $\Delta s$, and background plasma density, $n_\text {e}$, respectively. In (**c**) and (**d**), the effect of rephasing for different $\Delta s$ in (**c**), and $n_\text {e}$ in (**d**). For each of the parameters, $\Delta s$ or $n_\text {e}$ is kept constant, indicated by the text in each figure. The black dashed line and grey shaded area denote the mean divergence when Jet 2 is off (reference) and the r.m.s deviation, while the vertical error bars denote the r.m.s. deviation of $\theta _\text{x}$ and the peak energy, respectively. The horizontal bars indicate the accuracy of the separations for (**a**) and (**c**), and measured density in (**b**) and (**d**). The blue dashed-dotted line in each figure shows the theoretically expected divergence or energy boost for each parameter.

Depending on $\Delta s$ and $n_\text {e}$, the electron bunch will experience either a focusing- or a rephasing effect. In Fig. 3, an overview of the experimental results from one dataset is shown, representing typical data obtained from a tailored plasma density. In Fig 3a, the top image shows a reference electron spectrum with Jet 2 turned off. A slight tail of lower energies is observed, indicating that the optimum shock discussed in the previous section was not fully obtained. Fig. 3b shows that the spectrum in the reference case still reaches a peak energy close to 100 MeV. The two following spectra, shown in Fig. 3a, are obtained when Jet 2 is on. In the middle figure, a spectrum obtained with Jet 2 optimised for lensing is shown, while at the bottom, Jet 2 is optimised for rephasing. In Fig. 3b, these two regions are clearly observed.

For Jet 2 to efficiently act as a plasma lens, the separation, $\Delta s$, must be large enough such that the two jets are not interfering with each other; otherwise, disturbances due to colliding gas flows will appear. The absence of such disturbances is confirmed through shadowgrams recorded with a synchronised probe. In Figs. 4a and b, the transverse divergence, $\theta _\text {x}$, after propagation through Jet 2 is shown with respect to $\Delta s$ and $n_\text {e}$. The black dashed horizontal line and shaded grey area show the mean divergence measured from a set of reference spectra and their standard deviation. From Fig. 4a, a slight disturbance between the jets is observed for the shortest distance at $\Delta s = 2.7$ mm, which is the limit for a strong disturbance between the two jets. At shorter $\Delta s$, no consistent electron bunches in terms of particle energy and charge are obtained. However, as soon as $\Delta s$ increases, an optimum is obtained. In Fig. 4b, the dependence on the density of Jet 2 is shown at the optimum separation, $\Delta s = 3.7$mm. As expected for a plasma lens operating in the overdense regime^19^, the reduction of divergence at the optimum $\Delta s$ is insensitive to $n_\text {e}$.

In Figs. 4a and b, the blue dashed-dotted line shows the expected transverse bunch divergence with Jet 2 on, which is obtained by a simplified model, assuming a monochromatic bunch, no emittance growth, and that linear theory is applicable in this region. The focal length of the overdense plasma lens^19^ depends on the bunch density through $f=\gamma /(2\pi r_\text {e}n_\text {b}L$), where $r_\text {e}$ is the classical electron radius, *L* is the thickness of the lens, chosen as the FWHM of the density profile in Fig. 1b, and $\gamma$ is the electrons’ Lorentz factor. For a bunch with a Gaussian distribution, the bunch density is described by $n_\text {b} =N/((2\pi )^{3/2}\sigma _\text {x0}^2\sigma _\text {z})$, where *N* is the number of electrons contained within the bunch. The longitudinal size, $\sigma _\text {z}$, is assumed constant between Jet 1 and Jet 2, while the transverse size at the entrance to the plasma lens may be expressed as $\sigma _\text {x0}=\sigma _0\sqrt{1+((\Delta s -s_ \text {b})/\beta ^*)^2}$, where $\sigma _0$ is the electron bunch’s waist, obtained at a position $s_\text {b}$, and $\varepsilon _\text {n}$ is the normalised emittance. Note that $s_\text {b}$ is a position relative to $\Delta s$. Assuming $(\Delta s - s_\text {b}) \gg \beta ^*$, and $\beta ^* = \sigma _\text {0}^2\gamma /\varepsilon _\text {n}$, where the divergence is realised as $\theta _\text {x0}=\varepsilon _\text {n}/(\sigma _0\gamma )$, the transverse size at any position after Jet 1 is approximately $\sigma _\text {x0} \approx (\Delta s - s_\text {b}) \theta _\text {x0}$. For a thin lens, the divergence of the bunch under the influence of the plasma lens may be expressed as $\theta _\text {x} = \theta _\text {x0} - \sigma _\text {x0}/f$, and the above expression can therefore be combined to obtain the expected divergence for a given $\Delta s$:

1 $$\begin{aligned} \theta _\text {x}(\Delta s) = \theta _\text {x0}\left( 1-\frac{1}{\Delta s - s_\text {b}}\frac{\zeta NL}{\theta _{x0}^2\sigma _\text {z}\gamma }\right) , \end{aligned}$$

where $\zeta = r_\text {e}/(2\pi )^{1/2}$, and $s_\text {b}$ is used as a fitting parameter for the results in Figs. 4a and b, leading to $s_\text {b} \approx 0.6$ mm. $\theta _\text {x0}$ is the measured divergence of the electron bunch in the absence of the plasma lens. For our typical bunch charges of 15 pC and the divergence measured with Jet 2 off, the bunch density at the end of Jet 1 is estimated to be on the order of magnitude of $10^{17}$ cm$^{-3}$. In Fig. 4b, $n_\text {b}$ is constant since $\Delta s$ is kept at 3.7 mm, leading to focal length $f \approx 1$ cm. Thus, the increase in divergence with increasing $\Delta s$ originates primarily from the quadratic decrease of the bunch density.

When the two jets are brought together, such that $\Delta s < 3$ mm, the lens becomes less effective and the simple plasma lens model used above, deviates from the experimental data. As seen in Figs. 4c and d, Jet 2 is configured as a rephasing stage in this region, and a significant energy boost is observed. The dashed-dotted blue lines are theoretical estimations, where the energy boost is obtained as $\Delta E = eE_\text {z}L$. In both figures, the laser intensity is still high enough to excite a weak non-linear plasma wake ($a_0 \lesssim 1$). The electric field depends on $a_0$ and the plasma frequency, $\omega _\text {p} = \sqrt{n_\text {e} e^2/(\epsilon _0 m_\text {e})}$, through $E_\text {z}=a_0^2m_\text {e}c\omega _\text {p}/e$, and the trailing electrons can experience an accelerating field in Jet 2. The laser pulse intensity, $I(\Delta s)$ at $\Delta s$, affects $E_\text {z}$ through $a_0 = C \lambda \sqrt{I(\Delta s)}$, where $C = e/(\pi m_\text {e} c^{5/2} \sqrt{2\epsilon _0})$, and $\lambda$ is the centre wavelength of the laser pulse. Thus, the energy boost may be expressed as:

2 $$\begin{aligned} \Delta E(\Delta s, n_\text {e}) = C'\lambda ^2 I(\Delta s) \sqrt{n_\text {e}} L , \end{aligned}$$

where $C' = C^2ec\sqrt{m_\text {e}/\epsilon _0}$. Assuming a spatially and temporally Gaussian laser pulse, $I(\Delta s)$ of the diverging laser pulse at $\Delta s$ is $I(\Delta s)=I_0/(1+((\Delta s-s_\text {L})/z_R)^2)$, where $I_0$ is the laser pulse intensity in the focal spot, and $s_\text {L}$ the beam waist location, used as a fitting parameter in Figs. 4c and d. In Fig. 4c, $\omega _\text {p}$ is kept constant at the entrance of Jet 2 while $I(\Delta s)$ decreases with increasing $\Delta s$. Consequently, the effective $E_\text {z}$ decreases. At large $\Delta s$, the laser pulse will excite a weak wake, within which the electron bunch can drive its own wake, if $n_\text {b}$ is greater than the on-axis electron density. At this point, the electron bunch may instead experience a decelerating field from its own wake^28^. In Fig. 4d, $a_0$ at the entrance to Jet 2 is estimated from the fit to be 0.4. An energy boost is not observed until the density exceeds $10.5\times 10^{18}$ cm$^{-3}$, mainly attributed to the degree of plasma wave contraction. From the retrieved density profile, shown in Fig. 1b, the increase in density happens gradually until the maximum density of Jet 2 is reached, such that the electrons are kept in an accelerating phase at the upramp of Jet 2. As seen, the energy boost benefits from the increased plasma density, and the maximum energy gain is limited by the ability of the laser pulse to excite a plasma wave in Jet 2. However, at these high plasma densities, the wave contraction will eventually expose the electron bunch to the decelerating field of trailing wave periods. To fully describe the energy gain in Jet 2, the wakefield phase must be considered^21,29,30^.

In Fig. 3b, the charge transmission is about 20%. The charge loss may arise from plasma instabilities, coupling efficiency to the second jet, or inherent aberrations of the overdense plasma lens. However, the results presented here show that the beam quality can be significantly improved by combining several experimental techniques. The plasma tailoring offers great flexibility and robustness while keeping the accelerator compact. From Fig. 4, it is also realised that an intermediate configuration with both reduced electron bunch divergence and rephasing is attainable. In the future, a custom-made nozzle accommodating both a shock region (e.g., similar to the one used in ref^31^.) and a plasma lens/rephasing region with parallel gas flows is likely to improve the performance of the accelerator. Such a design would mitigate disturbances between the two jet configuration originating from the opposing gas flows. Further numerical investigation of the overdense plasma lens is needed to provide a full characterization for future optimisation.

## Summary and conclusion

We have experimentally demonstrated how a tailored plasma density distribution can provide stable acceleration of high-quality electron bunches. We confirm the reliability of shock-assisted ionisation injection and combine it with a second density region, providing an additional degree of freedom that allows further improvement of the electron bunch. This second region can be operated either as an overdense plasma lens or as a rephasing stage. Applications requiring high-quality electron bunches are a possibility^32^ since a fully LWFA-based beamline is straightforward to realise while relaxing the demands on the drive laser pulse. This type of scheme can also be used in an accelerator-radiator configuration to generate low-divergent x-rays^33,34^, or possibly as a compact LWFA-based free-electron laser^35–37^. The setup presented in this paper is robust and the results reproducible, while it allows maintaining a compact LWFA.

## Data Availability

Data is available from the corresponding author upon reasonable request.
